# Ecogeographical variable dataset for species distribution modelling, describing forest landscape in Latvia, 2017

**DOI:** 10.1016/j.dib.2022.108509

**Published:** 2022-08-05

**Authors:** Andris Avotins, Viesturs Kerus, Ainars Aunins

**Affiliations:** a Faculty of Biology, Department of Zoology and Animal Ecology, University of Latvia, Jelgavas Iela 1, Riga 1004, Latvia; b Latvian Ornithological Society, Skolas Iela 3, Riga 1010, Latvia

**Keywords:** Multi-scale forest landscape, Environmental heterogeneity, Raster layers, Ecological niche modelling

## Abstract

In this article, we provide access to, and information on the production of 42 ecogeographical variables (EGVs) used to describe the landscape of Latvia at three scales (local and two landscape). Layers are focused on the description of forest heterogeneity, but account for other ecosystems and land cover types as well. The more temporarily changing land use and land cover (LULC) types as forest and agricultural lands are described from 2017 databases. With most of the other LULC information was gathered from the topographic map (2016) at the scale of 1:10 000.

All the raster layers provided here are in the Latvian projected coordinate reference system (epsg:3059) with a grid cell size of 25 ha. Each layer provides quantitative information on the area, shape, and edge of habitat classes and additionally, age, time since the last forestry disturbance, relative soil humidity, relative soil richness etc. for forests. The three scales represent information from within 25 ha grid cell, and two radii – 1250 and 2500 m - around the centre of the grid cell.

## Specifications Table

**Table:** 

Subject	Biological sciences: Biodiversity
Specific subject area	In quantitative biological diversity studies, ecological niche and species distribution modelling play an important role. Here we are providing a dataset of ecogeographical raster layers necessary for such an analysis.
Type of data	Tables Raster layers (ASCII)
How the data were acquired	Data were acquired from nationally coordinated limited access and publicly available global databases that were further processed in geographic information systems (GIS). We used software ArcGIS 10.7 [2] with spatial analyst license in most of the data processing. For landscape ecology metrics, we used Fragstats 4.2.1 [3] and R 3.6 [4] with package “raster” [5] for file conversion, cell statistics and correlation analysis, and package “usdm” [6] for a multicollinearity analysis. We used Biomapper 4 [7] for a Box-Cox transformation [8].
Data format	Analysed
Description of data collection	We used information from all the main countrywide georeferenced databases describing land use and land cover. This information was acquired by the University of Latvia and the Latvian Ornithological society. Additionally, we used openly accessible information on tree cover and loss downloaded from www.globalforestwatch.org.
Data source location	Country: Latvia (whole inland territory) Dataset describes secondary data. Raw data are available from the following authorities: State Forest Service, upon application, provide forest stand level inventory database “Forest State Register”, email: pasts@vmd.gov.lv; Rural Support Service, upon application, provide agricultural field inventory data, email: pasts@lad.gov.lv; Latvian Geospatial Information Agency has paid service of providing topographic map layers at scale 1:10,000; Information on tree cover and its loss is freely available from https://www.globalforestwatch.org/
Data accessibility	Repository name: Mendeley Data [9] Data identification number: 10.17632/228vzwytth.1 Direct URL to data: https://data.mendeley.com/datasets/228vzwytth/1
Related research article	A. Avotins, V. Kerus, A. Aunins, 2022, National scale habitat suitability analysis to evaluate and improve conservation areas for a mature forest specialist species, Global Ecology and Conservation 38, e02218. 10.1016/j.gecco.2022.e02218[1]

## Value of the Data

• Information on the environment is crucial in studies of biological diversity, including biogeography and nature conservation. This dataset provides ready-to-use variables for such an analysis.
• Dataset is beneficial to everyone interested in studies of biological diversity and landscape ecology in Latvia. It ensures that variable preparation procedures are transparent and reduces time and computing constraints on the recreation of similar EGVs.
• The dataset provides a basis for species distribution modelling and conservation planning. By now it has been used only on a single species, but the EGVs provide information important for many more. Furthermore, as raw data used to prepare EGVs are not openly accessible and are not being archived, the dataset provides a reference for further investigations of LULC change.

## 1 Data Description

**Table 1** is a tab delimited (UTF-8 encoded) table with following fields:

Filename: name of file in folder Dataset;
Short_name: short variable name without file extension;
Full_name: explanatory name matching the description in the section “Experimental design, materials and methods”;
Mean_original: arithmetical mean of cell values;
SD_original: standard deviation of cell values;
BC_lambda: Box-Cox transformation parameter lambda;
BC_offset: Box-Cox transformation parameter offset;
Mean_BC: arithmetical mean of Box-Cox transformed cell values;
SD_BC: standard deviation of Box-Cox transformed cell values;
VIF: variance inflation factor values of Box-Cox transformed variables, if all of them are used simultaneously.

**Table 2** is a tab delimited (UTF-8 encoded) Pearson`s correlation coefficient matrix of Box-Cox transformed variables. Row and column names as in Table 1 field Short_name.

**Dataset** is an archived folder containing georeferenced (epsg: 3059; 500 m cell size) ASCII raster files (named as in Table 1 field Filename) of Box-Cox transformed ecogeographical variables. A total of 42 EGVs describe Latvian landscape at the analysis cell (500 m) and two radii – 1250 and 2500 m - around the centre of the grid cell. Full description of the dataset production procedure is given in the following section “Experimental design, materials and methods”. The mathethematical description and transformation parameters of every EGV are given in Table 1, the correlation matrix of all the variables are in Table 2 in the repository.

## 2 Experimental Design, Materials and Methods

### 2.1 Source Data

Information from all the main countrywide georeferenced databases describing land use and land cover (LULC) was acquired as source data to create ecogeographical variables (EGVs). The most important databases include the Forest State Register (forest stand-level inventory data; hereinafter FSR), agricultural field inventory data of the Rural Support Service (hereinafter RSS), and Latvian Geospatial Information Agency topographic map layers at scale 1:10,000 (hereinafter topographic map). This information was obtained by the University of Latvia project “The value and dynamic of Latvia's ecosystems under changing climate” and Latvian Ornithological Society projects preparing national conservation action plans for owls and woodpeckers from Nature Conservation Agency.

### 2.2 Input Data

All the mentioned databases include vector data that were rasterized to 25 m grid cell, ensuring pixels matching amongst layers, coordinate reference system (epsg: 3059) and the extent covering all national inland territory (all together – environments). These grids were used as input data to further process ecogeographical variables (EGVs) with 500 m raster grid cell (25 ha). Preparation of input data followed either of the following four workflows: presence/absence of habitat of interest (HOI; 1), quantitative condition of interest (COI; 2), distance to HOI (3) and combined landscapes (4).

Most of the **input data (1)** include information on the presence (value 1) or absence (value 0) of a HOI. These input data were created with the following procedure:

(1) features with the presence of HOI from all the source databases were merged into a single layer of geodatabase (or queried for processing if available in only one database) with a field containing value 1;
(2) presence layers (raster) were created with ArcGIS function “Feature to Raster”, ensuring environments;
(3) input data layer covering the whole country was created with ArcGIS function “Reclassify” with value 1 at presence location and value 0 at absences, ensuring environments.

**Input data (2)** containing quantitative COI were created as follows:

(1) features with the presence of COI from all the source databases were merged into a single layer of geodatabase (or queried for processing if available in only one database) with a field containing a value of the condition;
(2) input data, containing quantitative values were created with ArcGIS function “Polygon to Raster” with cell alignment type “Maximum combined area”, ensuring environments. This resulted in a layer with missing values that were treated individually for EGVs during their creation (see section “Ecogeographical variable raster layers”).

**Input data (3)** containing distance to HOI were created as follows:

(1) features with presence of HOI or COI from all the source databases were merged into a single layer of geodatabase (or queried for processing if available in only one database) with a field containing value 1;
(2) distance layers (raster) were created with ArcGIS function “Euclidean Distance” with “Plannar” distance method (all data are in epsg: 3059), ensuring environments.

Combined landscapes (**input data 4**) were created for traditional landscape ecology descriptions with Fragstats partial sampling analysis. As the dataset is focused on forests, we created a base landscape with “forests” (determined by the topographic map and FSR) on top of “other places with trees” (single trees, parks, alleys etc., determined by the topographic map), on top of “bushes” (bushes from RSS and topographic maps), on top of “wetlands” (bogs, mires and fens from FSR and topographic map), on top of “agricultural lands” (fields and grasslands from RSS and topographic maps and “other lands” from the topographic map), on top of “waterbodies” (from the topographic map) on top of “infrastructure and built-up” areas (from the topographic map). The base landscape was created with the ArcGIS tool “Combine” from input data (1) and reclassified (tool “Reclassify”), ensuring for environments in every step. To ensure sufficient area for information at landscape scale, the base landscape was 10 km larger than the national inland territory. Pixels with no values were classified as “others”.

Multiple landscapes (see section “Ecogeographical variable tables”) were created with different subclasses in “forests” and “agricultural lands”. We combined FSR on top of the topographic map in class “forests” and RSS data on top of the topographic map in class “agricultural lands”. Subclasses were added to the base landscape (from input data 1) with a custom Arc-Python script combining “Conditional” (“Con”) and “Logical” (“Equal To” and “Is Null”) tools from “Spatial Analyst Tools”, ensuring environments in every step. In cases of complete polygon or input data 1 overlap during sub-classification, higher priority was considered to classes of higher importance for the variable to be prepared.

### 2.3 Tree Species Groups

Tree species are grouped into three classes according to the code used in FSR:

**Broad-leaved**: Pedunculate Oak *Quercus robus* (FSR abbreviation – Oz, FSR code – 10), Ash *Fraxinus excelsior* (Os, 11), Small-leaved Lime *Tilia cordata* (L, 12), Elms *Ulmus laevis* and *U.glabra* (G, 16), Beech *Fagus sylvatica* (Ds, 17), Hornbeam *Carpinus betulus* (Sk, 18), Norway Maple *Acer platanoides* (K, 24), other oaks *Quercus* sp. (Ozc, 61), other limes *Tilia* sp. (Lc, 62), other maples *Acer* sp. (Kc, 63), other ashes *Fraxinus* sp. (Osc, 64), other Elms *Ulmus* sp. (Gc, 65), wallnuts *Juglans* sp. (R, 66), Horse Chestnut *Aesculus hippocastanum* (Z, 67).

**Boreal deciduous**: birches *Betula* sp. (B, 4), Black Alder *Alnus glutinosa* (M, 6), Aspen *Populus tremula* (A, 8), Grey Alder *Alnus incana* (Ba, 9), Balsam Poplar *Populus alba* (Pa, 19), Goat willow *Salix caprea* (Bl, 21), willows *Salix* sp. (Vī, 20), hybrid aspen *Populus tremuloides x P.tremula* (Ha, 68).

**Conifers**: Scots Pine *Pinus sylvestris* (P, 1), Norway Spruce *Picea abies* (E, 3), larches *Larix* sp. (Le, 13), other pines *Pinus* sp. (Pc, 14), other spruces *Picea* sp. (Ec, 15), Cedar *Pinus sibirica* (Cp, 22), firs *Abies* sp. (Be, 23), Yew *Taxus baccata* (I, 29), Douglas-Fir *Pseudotsuga* sp. (Du, 28).

In cases of stand-level classification, it was based on species with maximum stem volume proportion.

### 2.4 Legal Harvest Ages and Age Classes Per Dominant Tree Species

To create ecogeographical variables and interpret the results, we have used a forestry legislation-based approach for the age classification of forests. This approach considers tree species and growth conditions to define minimum rotation age (Table A.1). Final felling outside protected areas can be performed in younger stands if trees, on average in dominant (by volume) species, have reached minimum diameter (DBH).

**Table A.1:** Minimum rotation ages per site quality class in main tree species.

	Age (in years) per site quality class		
Dominant tree species	Highest	Medium	Lowest
Oaks	101	121	121
Pines and larches	101	101	121
Spruces, ashes, limes, elms, maples	81	81	81
Birches	71	71	51
Black Alder	71	71	71
Aspens	41	41	41

Currently, there is no minimum rotation age in Grey Alder. We used 35 years, as it is the age of the youngest stand registered as “full grown” in FSR. This was necessary for the harmonization of EGVs throughout forests.

Besides harvest age, we have referred to young stands. Although we used it as registered in FSR, typical classes are as follows: young stand – in coniferous trees, ashes and oaks – until 40 years, in Grey Alder – until 10 years, in other tree species – until 20 years. Stands are considered regenerated – young stands instead of clear-cuts – when coniferous trees are at least 0.1 metre and deciduous trees at least 0.2 m tall and in species dependant density:

Pines – 3000 trees/ha;
Oaks, ashes, elms, maples, beeches, hornbeams – 1500 trees/ha;
In other trees – 2000 trees/ha (for Black Alder, regenerating from roots, four shoots can be counted next to a single stump).

Besides, saplings must be equally spread, with no more than 20% of an area not considerable as regenerated.

### 2.5 Analysis Grid

Before the analysis and creation of all the EGVs, we experimented with different analysis grid sizes (100 m, 250 m, 500 m, 1000 m), estimating necessary computing resources and time and the capacity of the resulting variable to capture landscape heterogeneity, while providing a meaningful resolution. Due to computational limitations, we had to reject analysis using 100 and 250 m grids, while the 1000 m grid provided low heterogeneity and poor interpretability. Therefore, we performed the analysis in a regular grid with an edge of 500 m, covering the whole national inland territory. Grid (as a polygon with label points layer) was created with ArcGIS function “Create Fishnet” using the official national 1 km grid as a template. Polygons were used as a grid feature for zonal statistics in the creation of ecogeographical variables from input data (1–3). Points – as locations in landscape descriptions from input data 4.

Each grid cell has a unique identifier, matching polygon and point features. This identifier is saved in each ecogeographical variable table to be used for joining with the grid.

### 2.6 Ecogeographical Variable Tables

**Average aspen volume in 25** **ha landscape** – volume coefficient (25 m input raster values) of aspens and poplars *Populus* sp. (dominated by *Populus tremula*, codes 8, 18 and 68 in FSR) averaged over analysis cell (25 ha). ArcGIS function “Zonal Statistics as Table”.

**Average relative openness in 25** **ha landscape** – single countrywide landscape description created from all the input layers (25 m grid cells) with LULC class specific openness value. Arithmetic mean of classes per analysis cell (25 ha) calculated with ArcGIS function “Zonal Statistics as Table”. Class openness value:

- value 5: waters, bare soil and quarry areas from the topographic map, as well as winter and summer crops from RSS;
- value 4: permanent and cultivated grasslands from RSS, forest meadows from FSR and “other lands” from the topographic map, if not overlapping with other geometries;
- value 3: bogs, mires, heathlands and clear-cuts from the topographic map and FSR;
- value 2: gardens and fruit-tree plantations from the topographic map and RSS;
- value 1: forest stands lower than 5 m and bushlands from the topographic map and FSR;
- value 0: all other LULC.

**Average relative forest soil richness in 25** **ha landscape** – average (in 25 ha cell; ArcGIS function “Zonal Statistics as Table”) from classified input (25 m) forest growth conditions as used in FSR. Values per class: Sl (growth condition marker) = 1, Gs=2, Mr=3, Mrs=4, Pv=5, Ln=6, Nd=7, *Av*=8, Kv=9, Dm=10, Dms=11, Am=12, Km=13, Vr=14, Vrs=15, Db=16, As=17, Ks=18, Gr=19, Grs=20, Lk=21, Kp=22, Ap=23.

**Field and clear-cut edge with forests higher than 5** **m density in 490** **ha landscape** – a sum of pixels forming edges between classes (1 vs. 2):

(1) clear-cuts and forest meadows from FSR, “other LULC” from the topographic map, RSS registered areas, except fruit-tree plantations and bushes;
(2) Forests from the topographic map and tree stands (above 5 m) from FSR, and other trees from the topographic map.

The contrast between other classes is assumed to be 0, thus not participating in the sum. Analysis performed with Fragstats function “Contrast-Weighted Edge Density” with 1250 m radii around the analysis grid (25 ha) central 25 m cell.

**Field and clear-cut edge with forests higher than 5** **m density in 25** **ha landscape** – a sum of pixels forming edges between classes (1 vs. 2):

(1) clear-cuts and forest meadows from FSR, “other LULC” from the topographic map, RSS registered areas, except fruit-tree plantations and bushes;
(2) Forests from the topographic map and tree stands (above 5 m) from FSR, and other trees from the topographic map.

The contrast between other classes is assumed to be 0, thus not participating in the sum. Analysis performed with Fragstats function “Contrast-Weighted Edge Density” within a square with 500 m edge length.

**Edge of old (exceeding rotation age) forests with fields and clear-cuts in 490** **ha landscape** – sum (contrast weighted) of pixels (25 m) between forest stands at or exceeding harvest age registered in FSR and:

- with contrast value 1: waters, bare soil and quarry areas from topographic map, and arable lands (culture codes except: 620, 610, 710, 910, 911, 912, 914, 918, 919, 921, 922, 924, 926, 927, 928, 929, 931, 932, 933, 934, 935, 950, 952, 640) and grasslands (culture codes: 710, 720, 713, 714, 731, 732, 733, 734, 735, 736, 738, 739, 723, 724, 725, 726, 727, 728, 729) from RSS;
- with contrast value 0.75: forest meadows and clear-cuts from FSR and “other LULC” from topographic map;
- with contrast value 0.5: bogs, mires and heathlands from topographic map and FSR, gardens and fruit-tree plantations from topographic maps and RSS;
- with contrast value 0.25: young stands below 5 m and shrublands from FSR and topographic map.

The contrast between other classes is assumed to be 0, thus not participating in the sum. Analysis performed with Fragstats function “Contrast-Weighted Edge Density” with 1250 m radius around the analysis grid (25 ha) central 25 m cell.

**Edge of old (exceeding rotation age) forests with fields and clear-cuts in 25** **ha landscape** – sum (contrast weighted) of pixels (25 m) between forest stands at or exceeding harvest age registered in FSR and:

- with contrast value 1: waters, bare soil and quarry areas from topographic map, and arable lands (culture codes except: 620, 610, 710, 910, 911, 912, 914, 918, 919, 921, 922, 924, 926, 927, 928, 929, 931, 932, 933, 934, 935, 950, 952, 640) and grasslands (culture codes: 710, 720, 713, 714, 731, 732, 733, 734, 735, 736, 738, 739, 723, 724, 725, 726, 727, 728, 729) from RSS;
- with contrast value 0.75: forest meadows and clear-cuts from FSR and “other LULC” from topographic map;
- with contrast value 0.5: bogs, mires and heathlands from topographic map and FSR, gardens and fruit-tree plantations from topographic maps and RSS;
- with contrast value 0.25: young stands below 5 m and shrublands from FSR and topographic map.

The contrast between other classes is assumed to be 0, thus not participating in sum. Analysis performed with Fragstats function “Contrast-Weighted Edge Density” within a square with 500 m edge length.

**Average spruce volume in 25** **ha landscape** – volume coefficient (25 m input raster values) of spruces *Picea* sp. (dominated by Norway Spruce *Picea abies*, codes 3 and 15 in FSR) averaged over analysis cell (25 ha). ArcGIS function “Zonal Statistics as Table”.

**Area of young mixed forests in 490** **ha landscape** –mixed forest (conifers and deciduous trees with sum of volume coefficients >=3 each, per stand), registered as older than young stand and younger than rotation age in FSR, area. Analysis performed with Fragstats function “Class Area” with 1250 m radius around the analysis grid (25 ha) central 25 m cell.

**Area of old mixed forests in 490** **ha landscape** –mixed forest (conifers and deciduous trees with sum of volume coefficients >=3 each, per stand), registered as reached or exceeding rotation age in FSR, area. Analysis performed with Fragstats function “Class Area” with 1250 m radius around the analysis grid (25 ha) central 25 m cell.

**Area of young broad-leaved forests in 490** **ha landscape** – broadleaf forest (sum of volume coefficients >=5 per stand), registered as older than young stand and younger than rotation age in FSR, area. Analysis performed with Fragstats function “Class Area” with 1250 m radius around the analysis grid (25 ha) central 25 m cell.

**Area of old broad-leaved forests in 490** **ha landscape** – broad-leaved forest (sum of volume coefficients >=5 per stand), registered as reached or exceeding rotation age in FSR, area. Analysis performed with Fragstats function “Class Area” with 1250 m radius around the analysis grid (25 ha) central 25 m cell.

**Area of young boreal deciduous forests in 490** **ha landscape** –boreal deciduous forest (sum of volume coefficients >=7 per stand), registered as older than young stand and younger than rotation age in FSR, area. Analysis performed with Fragstats function “Class Area” with 1250 m radius around the analysis grid (25 ha) central 25 m cell.

**Area of old boreal deciduous forests in 490** **ha landscape** –boreal deciduous forest (sum of volume coefficients >=7 per stand), registered as reached or exceeding rotation age in FSR, area. Analysis performed with Fragstats function “Class Area” with 1250 m radius around the analysis grid (25 ha) central 25 m cell.

**Area of young coniferous forests in 490** **ha landscape** – coniferous forest (sum of volume coefficients >=8 per stand), registered as older than young stand and younger than rotation age in FSR, area. Analysis performed with Fragstats function “Class Area” with 1250 m radius around the analysis grid (25 ha) central 25 m cell.

**Area of old coniferous forests in 490** **ha landscape** – coniferous forest (sum of volume coefficients >=8 per stand), registered as reached or exceeding rotation age in FSR, area. Analysis performed with Fragstats function “Class Area” with 1250 m radius around the analysis grid (25 ha) central 25 m cell.

**Average forest (higher than 5** **m) patch depth** – average per 25 ha analysis cell of Euclidean distances from inside forest >= 5 m high to its nearest edge (25 m input raster). Aggregation with ArcGIS function “Zonal Statistics as Table”.

**Area of oligotrophic forests on mineral soils in 490** **ha landscape** – forest types according to the national classification (typology) “Sl”, “Mr”, “Mrs” and “Gs” area. Analysis performed with Fragstats function “Class Area” with 1250 m radius around the analysis grid (25 ha) central 25 m cell.

**Area of mesotrophic forests on mineral soils in 490** **ha landscape** – national forest of types “Ln”, “Dm” and “Dms” area. Analysis performed with Fragstats function “Class Area” with 1250 m radius around the analysis grid (25 ha) central 25 m cell.

**Area of eutrophic forests on mineral soils in 490** **ha landscape** – national forest of types “Vr”, “Vrs”, “Gr” and “Grs” area. Analysis performed with Fragstats function “Class Area” with 1250 m radius around the analysis grid (25 ha) central 25 m cell.

**Area of oligotrophic forests on peat soils in 490** **ha landscape** – national forest of types “Pv” and “Nd” area. Analysis performed with Fragstats function “Class Area” with 1250 m radius around the analysis grid (25 ha) central 25 m cell.

**Area of eutrophic forests on peat soils in 490** **ha landscape** – national forest of types “Db” and “Lk” area. Analysis performed with Fragstats function “Class Area” with 1250 m radius around the analysis grid (25 ha) central 25 m cell.

**Area of oligotrophic forests on drained soils in 490** **ha landscape** – national forest of types “*Av*”, “Am”, “Kv” and “Km” area. Analysis performed with Fragstats function “Class Area” with 1250 m radius around the analysis grid (25 ha) central 25 m cell.

**Area of eutrophic forests on peat drained in 490** **ha landscape** – national forest of types “As”, “Ap”, “Ks” and “Kp” area. Analysis performed with Fragstats function “Class Area” with 1250 m radius around the analysis grid (25 ha) central 25 m cell.

**Maximum largest tree diameter in 25** **ha landscape** – largest tree diameter per stand (as registered in FSR) in input (25 m) data. Maximum value per analysis grid (25 ha) calculated with ArcGIS function “Zonal Statistics as Table”.

**Average largest tree diameter in 25** **ha landscape** – largest tree diameter per stand in input (25 m) data. Mean value per analysis grid (25 ha) calculated with ArcGIS function “Zonal Statistics as Table”.

**Average relative soil humidity in 25** **ha landscape** – national forest growth classes coded to form a relative humidity gradient, with dry mineral soils=1, drained mineral soils=2, drained peat soils=3, humid mineral soils=4 and peat soils=5 in 25 m input raster. Average value per analysis grid (25 ha) calculated with ArcGIS function “Zonal Statistics as Table”.

**Average relative understorey density in 25** **ha landscape** – national forest site types recoded (in 25 m input raster) to represent a relative (experts opinion) gradient: Sl=1,Gs=2,Mr=3,Mrs=4,Ln=5,Pv=6,Dm=7,Dms=8,Nd=9,*Av*=10,Kv=11,Am=12,Km=13,Vr=14,Vrs=15,Db=16,Gr=17,Grs=18,As=19,Ks=20,Ap=21,Kp=22,Lk=23. Average value calculated with ArcGIS function “Zonal Statistics as Table”.

**Average broad-leaved tree volume in 25** **ha landscape** – sum of volume coefficients (per stand in 25 m input raster) of broad-leaved trees (abbreviations: Oz, Os, L, G, Ds, Sk, K, Ozc, Lc, Kc, Osc, Gc) averaged over analysis cell (25 ha). ArcGIS function “Zonal Statistics as Table”.

**Average pine volume in 25** **ha landscape** – volume coefficient (25 m input raster values) of pines *Pinus* sp. (dominated by Scots Pine *Pinus sylvestris*, codes 1, 14 and 22 in FSR) averaged over analysis cell (25 ha). ArcGIS function “Zonal Statistics as Table”.

**Average boreal deciduous tree volume in 25** **ha landscape** – sum of volume coefficients (per stand in 25 m input raster) of boreal deciduous trees (abbreviations in FSR: B, M, A, Ba, Pa, Vi, Bl) averaged over analysis cell (25 ha). ArcGIS function “Zonal Statistics as Table”.

**Average tree size in 25** **ha landscape** – largest stand-level size (hight multiplied by diameter) of a tree group as input (25 m) raster value. Average per analysis grid (25 ha) calculated with ArcGIS function “Zonal Statistics as Table”.

**Average time since the last forestry (tree cutting or planting) disturbance in 25** **ha landscape** – time since the last registered activity in FSR until 2017, if no activity is registered, the time since the last event is assumed to be equal to the age of the stand. Average per analysis grid (25 ha) calculated with ArcGIS function “Zonal Statistics as Table”.

**Time since the last forestry (tree cutting or planting) disturbance in 25** **ha landscape** – time since the last registered activity in FSR until 2017, if no activity is registered, the time since the last event is assumed to be equal to the age of the stand. Minimum per analysis grid (25 ha) calculated with ArcGIS function “Zonal Statistics as Table”.

**Area of young forests in 1900** **ha landscape** – stand, registered as older than young stand and younger than rotation age in FSR, area. Analysis performed with Fragstats function “Class Area” with 2500 m radius around the analysis grid (25 ha) central 25 m cell.

**Area of old forests in 1900** **ha landscape** – stand, registered as reached or exceeding rotation age in FSR, area. Analysis performed with Fragstats function “Class Area” with 2500 m radius around the analysis grid (25 ha) central 25 m cell.

**Area of tree cover in 25** **ha landscape** – from cells (www.globalforestwatch.org) with tree cover (value >=1) subtracted cells with tree cover loss until 2017 and added cells with tree cover gain until 2012, everything recoded to 0/1 with ArcGIS function “Raster calculator”. Result was resampled to 5 m pixel size with method “NEAREST” in ArcGIS function “Resample” and back to 25 m matching other input data with “maximum” method in ArcGIS function “Aggregate”. Sum of values per analysis grid (25 ha) calculated with ArcGIS function “Zonal Statistics as Table”.

**Maximum forest stand age as a fraction of rotation age in 25** **ha landscape** – each forest stand has its rotation age, which is based on the dominant species, growth speed and protection regime (in the case of grey alder *Alnus incana*, 35 years assumed), then from FSR registered stand age is rotation age subtracted (younger stands form negative values) and the obtained value is divided by stand specific rotation age to standardize between different stands. Maximum value per analysis grid (25 ha) calculated with ArcGIS function “Zonal Statistics as Table”.

**Average forest stand age as a fraction of rotation age in 25** **ha landscape** – each forest stand has its rotation age, which is based on the dominant species, growth speed and protection regime (in the case of grey alder *Alnus incana*, 35 years assumed), then from FSR registered stand age is harvest age subtracted (younger stands form negative values) and the obtained value is divided by stand specific rotation age to standardize between different stands. Average value per analysis grid (25 ha) calculated with ArcGIS function “Zonal Statistics as Table”.

**Area of fields and grasslands in 25** **ha landscape** – fields registered in RSS with cultures excluding fruit-trees and bushlands, supplemented with forest meadows from FSR and “other lands” from the topographic map (if not containing other geometries). Sum of 25 m input pixels per analysis grid (25 ha) with ArcGIS function “Zonal Statistics as Table”.

**Area of waterbodies in 25** **ha landscape** – sum of 25 m input pixels containing waterbodies registered in the topographic map, calculated with ArcGIS function “Zonal Statistics as Table”.

**Area of wetlands in 25** **ha landscape** - – sum of 25 m input pixels containing bogs and mires registered in FSR and topographic map, calculated with ArcGIS function “Zonal Statistics as Table”.

### 2.7 Ecogeographical Variable Raster Layers

Every table containing a description of ecogeographical variables was joined to the attribute table of the polygon grid based on the grid cell's unique identifier. This introduced missing values in some locations in EGVs from input data 2 and 4. Missing values were substituted with the lowest reasonable value, which in most cases was 0, representing no edge/no area/no volume. Treatment with value 0 was also applied to variables meaningful to forests in areas outside forests (if the analysis grid cell contains any forest pixel in input data, the value is used, missing are present only outside forests), resulting in no forest soil humidity/ no understorey density etc. The only exceptions are two variables representing relative forest age (maximum and average forest stand age as a fraction of harvest age in 25 ha landscape), when the value “−1″ was used. In those cases, value 0 would mean that all the forest stands are at the age of harvest, while “−1″ is equivalent to no age, therefore, no forest.

We used a custom Arc-Python script to join the tables to the attribute table of the georeferenced grid, then treated missing values according to the previously described procedure (combining functions “Con” and “Is Null” from “Spatial Analyst Tools”) and exporting GeoTiff, while ensuring for environments. Whereas transformation from GeoTiff to ASCII file extension was carried out in software R package “raster”.

Each missing-value-treated and georeferenced EGV table was then transformed into a GeoTiff raster layer. These layers were Box-Cox transformed [8], due to strongly skewed distributions (description in Table 1 in repository). Transformed layers were saved as separate GeoTiff files and transformed into ASCII files (available in the archived folder “Dataset” in the repository). Variable pairwise Pearson`s correlation coefficients are in Table 2 in the repository.

## Ethics Statements

Our work does not involve human subjects, animal experiments or data collected from social media platforms.

## CRediT authorship contribution statement

**Andris Avotins:** Writing – original draft, Conceptualization, Methodology, Data curation, Formal analysis. **Viesturs Kerus:** Conceptualization, Data curation, Writing – review & editing. **Ainars Aunins:** Conceptualization, Data curation, Methodology, Supervision, Writing – review & editing.

## Declaration of Competing Interest

The authors declare that they have no known competing financial interests or personal relationships that could have appeared to influence the work reported in this paper.

## Data Availability

Ecogeographical variable dataset for species distribution modelling, describing forest landscape in Latvia, 2017 (Original data) (Mendeley Data).
